# Current utilization trend of immortalized mast cell lines in allergy research: a systematic review

**DOI:** 10.1007/s12026-024-09562-w

**Published:** 2025-01-21

**Authors:** Ashley Jia Wen Yip, Yu Zhao Lee, Audrey Siew Foong Kow, Carisa Su-Ann Wong, Ming-Tatt Lee, Chau Ling Tham, Ji Wei Tan

**Affiliations:** 1 School of Science, Monash University Malaysia, Jalan Lagoon SelatanSubang Jaya, 47500 Bandar Sunway, Selangor Malaysia; 2 Faculty of Medicine and Health Sciences, UCSI University, Cheras, 56000 Kuala Lumpur, Malaysia; 3 Faculty of Pharmaceutical Sciences, UCSI University, Cheras, 56000 Kuala Lumpur, Malaysia; 4 Graduate Institute of Pharmacology, College of Medicine, National Taiwan University, Taipei, 10051 Taiwan; 5 Department of Biomedical Science, Faculty of Medicine and Health Sciences, Universiti Putra Malaysia, 43400 Serdang, Selangor Malaysia; 6 Natural Medicine and Product Research Laboratory (NaturMeds), Institute of Bioscience, Universiti Putra Malaysia, 43400 Serdang, Selangor Malaysia

**Keywords:** Allergy, Immortalized mast cells, In vitro, HMC-1, LAD2, RBL-2H3

## Abstract

Today, in the modern world, allergic diseases, also described as atopic allergies, are classified as a type of multifactorial disorder due to the complex interplay between genetics, environment, and socioeconomic factors that influence the disease’s manifestation, severity, and one’s predisposition to allergic diseases. It is undeniable that many reported studies have pointed out that the mast cell is one of the leading key players involved in triggering an allergic reaction. To improve our understanding of the molecular and cellular mechanisms underlying allergy, various mast cell lines have been employed in vitro to study the pathogenesis of allergic diseases for the past decades. However, there is no consensus on many fundamental aspects associated with their use, such as the effects of culture media composition and the type of inducer used for cell degranulation. As the standardization of research protocols and disease models is crucial, we present the outcome of a systematic review of scientific articles using three major immortalized in vitro mast cell lines (HMC-1, LAD2, and RBL-2H3) to study allergy. This systematic review described the cell source, culture conditions, inducers used for degranulation, and mediators released for examination. We hope that the present systematic review may help to standardize the use of immortalized in vitro mast cell lines in allergy research and serve as a user’s guide to understand the fundamental aspects of allergy as well to develop an effective allergy therapy in the future for the betterment of human good health and wellbeing.

## Introduction

Humans have battled with allergies for decades. The concept of allergies was first introduced in the early twentieth century by an Austrian scientist named von Pirquet, describing that an allergy occurs when exogenous substances (allergens) induce a change in reactivity in an individual’s immune system, leading to hypersensitive reactions [1]. Typical allergens are found in a wide range of environmental substances varying in their nature and source, including food allergens, aeroallergens like pollen, mites, and dust, as well as chemical allergens like dyes, creams and skincare products [2]. Common allergic diseases include atopic eczema (dermatitis), rhinitis, allergic asthma, and drug and food allergies. Allergic diseases are considered a worldwide severe health issue, and their prevalence comprises a substantial percentage of the population. Rhinitis and food allergies affect 10–30% and 8% of the population worldwide, respectively, while skin allergies such as eczema have a lifetime prevalence of 20% worldwide [2]. Allergy symptoms range from mild, such as itchiness, hives, watery eyes, and a runny nose, to life-threatening outcomes, depending on the hyperreactivity of the immune system. The lethal and exaggerated allergic reaction known as anaphylaxis is the primary cause of death in allergic patients [3].

Currently, the most well-known curative treatment for IgE-mediated allergies is allergen-specific immunotherapy (AIT). This therapy involves subcutaneous administration of gradually increasing quantities of a patient’s corresponding allergen until an ideal dose capable of stimulating immune tolerance toward the allergen is achieved [4]. Immunologic improvements in patients subjected to AIT are associated with the production of T regulatory cells that induce the anti-inflammatory cytokine interleukin (IL)−10. This causes an early decrease in mast cells, basophil activation, and the subsequent reduction of inflammatory mediators such as histamine [5]. Despite the efficacy of AIT, the development of immune tolerance in patients is still an evolving area. Other short-lived first-line treatments widely used consist of inhalation of corticosteroids, β-adrenergic agonists, and leukotriene modifiers in allergic asthma, or the avoidance of the food allergen and treatment with antihistamines for mild symptoms of food allergies are available [6, 7]. However, these forms of treatment merely alleviate allergy symptoms rather than target the underlying pathology of the disorder.

The use of in vitro mast cell models may be able to answer and resolve some of the issues faced with current treatment. Mast cells have been considered the primary effector cells in allergic reactions, and as a result, they have become attractive candidates in the study of allergenicity and sensitization mechanisms. Mast cells originate from multipotent hematopoietic stem cells that are mainly distributed in blood vessels located at the host-environment interface, such as the skin, airways, and gastrointestinal tract. Their localization in the body makes them one of the first immune cells to interact with incoming allergens [3]. As described previously, mast cells play a central role during an allergic reaction. As mast cells are packed with the high-affinity IgE receptor FcεRI, binding of allergen-specific IgE stimulates mast cell degranulation releases prestored proinflammatory mediators such as histamine, serotonin, and proteases as well as de novo synthesis of inflammatory mediators such as leukotriene and prostaglandins [8]. This surge in the excessive release of such mediators rapidly triggers anaphylactic shocks. As mast cells differentiate in the peripheral tissues from progenitor cells in the bone marrow, CD34^+^ myeloid progenitor cells, derived from buffy coats, cord blood, or bone marrow, have been used as the primary source for generating mast cells in vitro [9]. However, in vitro research using human mast cells poses several challenges, such as low proliferative activity and the differentiation steps occurring physiologically in tissues. These are time-consuming, difficult, and expensive to recapitulate in vitro [10]. As such, several commercial human mast cell lines have been generated, such as the HMC-1 (human mast cell line 1), LAD2 (laboratory of allergic diseases 2), and LUVA (Laboratory of University of Virginia) as well as rodent mast cell lines such as the RBL-2H3 (rat basophilic leukemia-2H3) cell line which are routinely used as in vitro allergy models depending on their specific advantages and limitations [11]. Although no model has been able to fully replicate human mast cell phenotypes, given the right culturing conditions and experimental setup, each model may possess some benefit over the selection of others.

To our knowledge, there is relatively limited data on large-scale tabulated data regarding the usage of mast cell line models. Hence, this paper systematically reviews three common immortalized mast cell line models (HMC-1, LAD2, and RBL-2H3) used in allergy-related studies for their culturing conditions, types of inducers used, and inducing conditions. We also draw comparative tabulations and reasonings on the mast cell line used on their laboratory conditions and experimental purposes. The review seeks to provide researchers with details on the characteristics and mechanisms of each mast cell model to aid in the proper selection of models for future studies.

## Methods

### Search strategy

Relevant articles were identified from two databases (Web of Science and ScienceDirect) using the keywords: allergic inflammation AND mast cell activation. All reported research studies that use in vitro mast cells were included in this systematic review. Filter was applied to include research articles in English that were published from 2018 to 2023. This study was conducted according to the Preferred Reporting Items for Systematic Reviews and Meta-analysis (PRISMA) 2020 guidelines. The last search for relevant articles in all databases was performed on 31st of January 2024.

### Eligibility criteria

For this systematic review, the inclusion criteria are (1) in vitro mast cell studies, (2) secondary cell line culture studies, and (3) allergy-related studies. Only published research articles from 2018 to 2023 were included. On the other hand, the exclusion criteria are (1) animal work that does not involve any in vitro mast cell studies, (2) only involves primary cell line culture studies, and (3) full-text not accessible.

### Study appraisal and selection

**Fig. 1:**
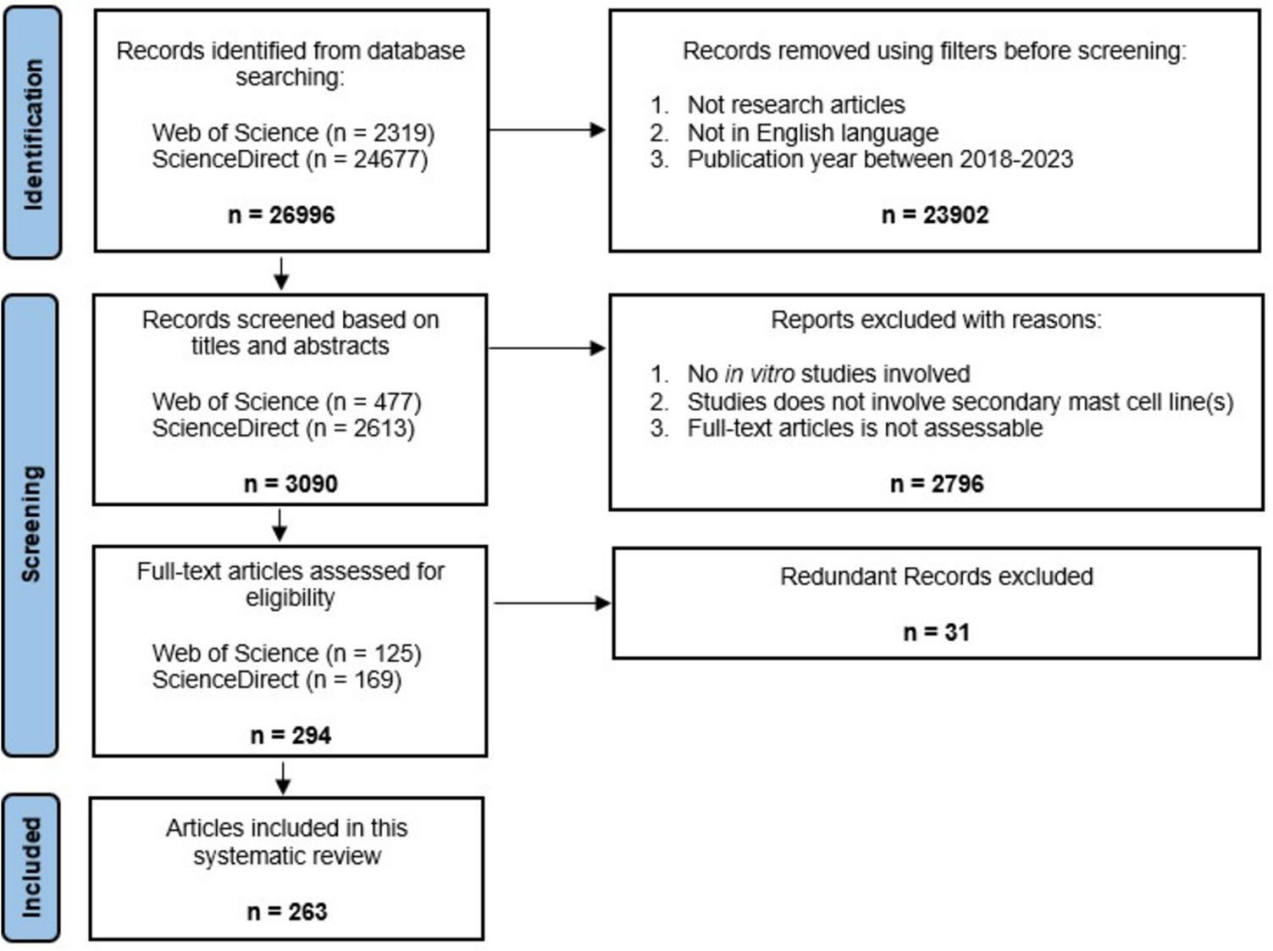
Flowchart showing the selection process of articles according to the PRISMA 2020 Statement. From the initial 26,996 non-redundant articles, 263 articles were included in the systematic review

All the articles obtained from the databases using the specific keywords were organized according to their titles, and duplicates were identified by the same title, authors, and year of publication. The redundant studies were removed, and the remaining articles were screened using the pre-defined eligibility criteria. The title and abstract of each article were first independently assessed by two reviewers. Those that matched the eligibility criteria were then subjected to full-text screening to determine their relevance further. Disagreements between the two reviewers throughout the screening process were resolved by consensus, and the reasons for excluding the articles were recorded (Fig. 1).

## HMC-1 cell line

The human mast cell-1 line (HMC-1) is a well-known model used in allergic and inflammatory disease studies. It was established from a mast cell leukemia patient, entailing a dedifferentiated, spindle-shaped hypogranular appearance [9, 12]. While it is considered an immature mast cell, the HMC-1 cell line shares a phenotype akin to human mast cells (HuMC). The expression of mast cell-associated markers (i.e., histamine, heparin, tryptase, and c-kit receptor) and a similar cell surface antigen profile render the HMC-1 cell line a strong candidate in allergy research [13, 14]. HMC-1 can be further subdivided into HMC-1.1 (HMC^560^) and HMC-1.2 (HMC^560, 816^), depending on the location of the mutation in the c-kit receptor. HMC-1.1 possesses a substitution of glycine-560 to valine (V560G), while HMC-1.2 contains the V560G mutation and another substitution of valine-816 to aspartate (D816V) [15, 16]. These mutations in the c-kit receptor cause dysregulation in c-kit receptor expression, thus resulting in the survival of HMC-1 cell lines independent of stem cell factor (SCF), which is essential for mast cell proliferation, chemotaxis, activation, differentiation, and survival [17, 18]. This allows HMC-1 cells to be highly favorable in in vitro mast cell research as they have a higher proliferative rate than other mast cell lines. The doubling time of the HMC-1 cell line (1–3 days) is described to be tenfold faster than the LAD2 cell line (10–14 days) [9, 19]. In addition, when compared between the two variant sublines, HMC-1.2 exhibits a higher proliferative rate than HMC-1.1 due to the presence of D816V mutation [20]. This mutation leads to constitutive tyrosine kinase activation of the Kit receptor, which causes a higher proliferation rate than the HMC-1.1, which does not have the same mutation point [21].

Despite sharing a phenotype similar to HuMCs, the HMC-1 cell line is dissimilar to mature HuMCs. HMC-1 cell line has a low expression of mature HuMCs markers (i.e., tryptase and chymase), except for c-kit and histamine, representing immature malignantly transformed mast cells [22]. The binding of IgE to the high-affinity IgE receptor FcεRI is crucial for the activation and degranulation of mast cells [23]. As such, the lack in surface expression of FcεRI in the HMC-1 cell line requires the usage of a physiological stimulus, such as phorbol myristate acetate (PMA), calcium ionophore (CI), and compound 48/80 (C48/80) [24]. Additionally, Mas-Related G-protein coupled Receptor X2 (MRGPRX2) is known to induce mast cell degranulation, contributing to pseudo-allergic reactions caused by small molecule drugs. The HMC-1 cell line, however, expresses lower levels of MRGPRX2 and poor degranulation activity than the LAD2 cell line and HuMCs. It is noted that latrunculin-B can be used to prompt MRGPRX2-mediated degranulation [9]. Overall, the shortcomings of the HMC-1 cell line may potentially limit the insights available to mast cell activation studies. Yet, the high proliferative rate and stable phenotype render them a feasible model for extensive in vitro studies.

### Cell source and culture conditions of HMC-1 cell line

**Fig. 2:**
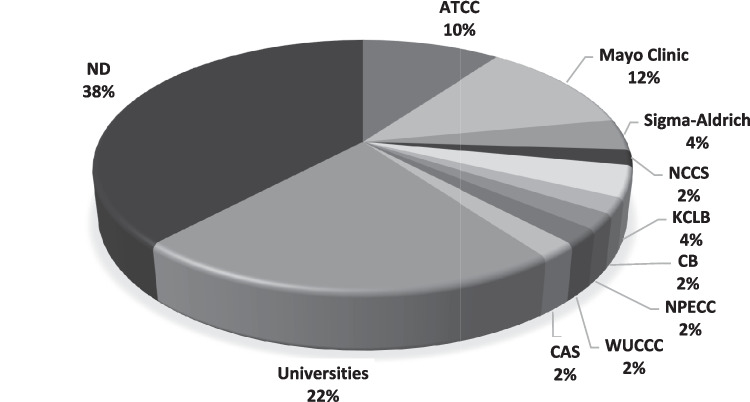
Sources of the reported HMC-1 cell line. The proportion of articles using a particular source is indicated in the main text. ATCC, American Type Culture Collection; NCCS, National Centre for Cell Science; KCLB, Korean Cell Line Bank; CB, Cellcook Biotechnology; NPECC, National Platform of experimental cell resources; WUCCC, Wu-Han University Cell Collection Center; CAS, Chinese Academy of Sciences; ND, no data

The HMC-1 cell line is often sourced from cell banks (20 of 50), with the most provided by Dr. Joseph H. Butterfield from Mayo Clinic (6 of 50), followed by American Type Culture Collection (ATCC) (5 of 50), Sigma-Aldrich (2 of 50), and Korean Cell Line Bank (KCLB) (2 of 50). Other cell banks include the Cellcook Biotechnology (CB), the National Platform of experimental cell resources (NPECC), the National Centre for Cell Science (NCCS), Wu-Han University Cell Collection Center (WUCCC), and the Chinese Academy of Sciences (CAS). In addition, HMC-1 cell lines from 11 out of 50 publications were gifted by other institutions, with the most from Eiichi Morri Osaka University (4 of 50). Following that, Prof. Jae-Young Um from KyungHee University and Prof. Jong-Sik Jin from Jeonbuk University contributed 2 out of 50 publications each. Other institutes include Hoseo University (Prof. Hyun-Ja Jeong), Second Military Medical University (Zhi Liang Yu), and Sangji University. However, the authors did not report the source of their gifted HMC-1 cell line. Finally, 19 of the shortlisted publications did not specify the cell origin. The sources of HMC-1 are summarized in Fig. 2.

The growth medium composition used for HMC-1 cell line propagation varies among publications. Sigma-Aldrich and MERCK recommend that the HMC-1 cell line be maintained in IMDM supplemented with 10% fetal bovine serum (FBS), 1.2 mM α-thioglycerol, and 1 × penicillin/streptomycin in a 37 °C humidified environment with 5% CO_2_. In actual protocols employed in the publications, most of the articles maintain the HMC-1 cell line in IMDM (43 of 50), followed by RPMI-1640 (5 of 50), DMEM (1 of 50), and IMEM (1 of 50). In addition, the media was supplemented with 10% FBS (45 of 50), penicillin/streptomycin (44 of 50), 2 mM L-glutamine (4 of 50), monothioglycerol (3 of 50), α-thioglycerol (2 of 50), 10% fetal calf serum (FCS) (2 of 50), 2-mercaptoethanol (1 of 50), amphotericin B (1 of 50), and sodium bicarbonate (1 of 50). Table 1 summarizes the culture medium and conditions to grow HMC-1 cells.

**Table 1:** Detailed information on the type of basal media, supplements used, and their respective number of articles for culturing HMC-1 cells

Basal media		Supplements			
		Serum		Others	
Name	# articles	Name	# articles	Name	# articles
IMDM	43	10% FBS	45	Penicillin/streptomycin	44
RPMI-1640	5	10% FCS	2	L-glutamine	4
DMEM	1			Monothioglycerol	3
IMEM	1			α-Thioglycerol	2
				2-mercaptoethanol	1
				Amphotericin B	1
				Sodium bicarbonate	1

*IMDM*, Iscove’s Modified Dulbecco’s Medium; *RPMI-1640*, Roswell Park Memorial Institute-1640; DMEM, Dulbecco’s Modified Eagle Medium; *IMEM*, improved minimum essential media; *FBS*, fetal bovine serum

The choice of medium and composition is crucial to provide an optimal cell growth and survival environment. IMDM, a highly enriched synthetic medium, is often recommended for rapidly proliferating and high-density cell lines. While no studies have reported the correlation between medium composition and the growth of HMC-1 cells, evidence shows that DMEM and RPMI-1640 media can affect the growth and differentiation of several cell lines [25, 26]. The medium’s excess or lack of calcium ion (Ca^2+^) and inorganic phosphate (Pi) may attenuate the differentiation of several cell lines. The concentrations of 1.8 mM and 0.09 mM of Ca^2+^ and Pi, respectively, are optimum for cell proliferation [27]. L-glutamine is supplemented to the medium as an energy source for rapidly dividing cell lines [28]. The degradation of L-glutamine to ammonia may be toxic to the cells, where 2 to 3 mM is sufficient to reduce cell growth. Yet, such occurrence is dependent on the cell line [29]. In addition, there are contradictory reports on the effects of supplementing media with L-glutamine on cytokine release. Coëffier et al. (2001) demonstrated the reduction in proinflammatory cytokines (IL-6 and IL-8) from human intestinal mucosa by glutamine via a post-transcriptional pathway [30]. Glutamine has also decreased the expression of leukotriene C_4_, monocyte chemoattractant protein (MCP), macrophage inflammatory protein (MIP)−1β, tumor necrosis factor-alpha (TNF-α), interleukin (IL)−15, and IL-18 in human intestinal mast cells and lobectomy patients [31, 32]. In contrast, several publications observed an increase in Th1 cytokines (IL-2 and IFN-γ) in PMACI-treated intestinal intraepithelial lymphocytes by glutamine [33]. Similarly, IL-1 and IL-10 were upregulated in glutamine-treated lobectomy patients [32]. Other supplements, such as sodium pyruvate in IMDM, have been shown to impair cytokine production by inhibiting inflammatory signaling pathways [34].

### Inducers and mediators release for HMC-1 cells

As the HMC-1 cell line lacks the expression of FcεRI, an external stimulus is required to induce mast cell degranulation. The combination of PMA (25 of 50) and CI is widely used (28 of 50). Other inducers include OVA, IL-33, TSLP, protein kinase activator C, RANKL, LPS, and histamine. However, three of the 50 publications did not specify the type of inducers used. The induction time implemented by most studies falls within 5 to 8 h (h) (23 of 50), followed by an hour and below (20 of 50), within 13 to 24 h (16 of 50), 2 to 4 h (13 of 50), and 9 to 12 h (2 of 50). However, four publications did not indicate the induction time. The concentration of cytokines secreted can characterize the degranulation of mast cells. Many of the publications studied the release of TNF-α (29 of 50), IL-6 (27 of 50), IL-1β (20 of 50), histamine (17 of 50), TSLP (14 of 50), and IL-8 (12 of 50). Table 2 summarizes our analysis of the type of inducers, induction time, and type of mediators often studied by researchers using HMC-1 cells.

**Table 2:** Detailed information on the number of articles for the type of inducers, their induction time, and the type of mediators studied in HMC-1 cell activation

Type of inducers		Induction		Type of mediators	
Name	# articles	Time	# articles	Name	# articles
PMA	25	< 1 h	20	TNF-α	29
CI	28	2–4 h	13	β-Hexosaminidase	7
Compound 48/80	3	5–8 h	23	Histamine	17
OVA	3	9–12 h	2	IL-1β	20
IL-33	3	13–24 h	16	IL-1RA	1
TSLP	3	Unknown	4	IL-2	2
PKC activator	2			IL-4	7
RANKL	2			IL-5	4
LPS	2			IL-6	27
Histamine	2			IL-7RA	1
DNP-HAS	1			IL-8	12
EGCG	1			IL-10	7
IgD	1			IL-13	6
IgE	1			IL-17	2
PFC	1			IL-18	2
BPA	1			IL-21	1
DMSO	1			IL-23	1
Bisdemethoxycurcumin	1			IL-31	1
Thrombin	1			IL-33	1
Par1 agonist	1			IFN-γ	3
Par4 agonist	1			IgE	1
Par1 inhibitor	1			IgG1	1
Par4 inhibitor	1			IgG2a	1
ERK1/2 inhibitor	1			LTB4	2
P38 inhibitor	1			LTC4	1
JNK inhibitor	1			PGD2	1
*Lactococcus lactis*	1			TSLP	14
Unknown	3			RANTES	1
				VEGF	1
				TGFβ1	1
				CXCL 10	1
				Tryptase	3
				Caspase 1	9
				Calcium ions	6

*PMA*, phorbol 12-myristate 13-acetate; *CI*, calcium ionophore; *OVA*, ovalbumin; *TSLP*, thymic stromal lymphopoietin; *RANKL*, receptor activator of NF-κβ ligand; *LPS*, lipopolysaccharide; *DNP*, 2, 4-dinitrophenol; *EGCG*, epigallocatechin gallate; *PFC*, perfluoroalkyl compounds; *BPA*, bisphenol A; *DMSO*, dimethylsulfoxide; *TNF*, tumor necrosis factor; *RA*, receptor antagonist; *LTB4*, leukotriene B4; *LTC4*, leukotriene C4; *PGD2*, prostaglandin D2; *RANTES*, regulated upon activation, normal T cell expressed and secreted; *PKC*, PKC; *VEGF*, vascular endothelial growth factor; *CXCL*, chemokine (C-X-C motif) ligand 1; *ND*, no data

Mast cell activation depends on the cross-linking of IgE antibodies on the FcεRI and the subsequent signal transduction cascade, including intracellular Ca^2+^ mobilization, influx, and protein kinase C (PKC) activation. As such, the combination of PMACI is a strong candidate as an inducer owing to its ability to enhance Ca^2+^ influx and activation of MAP kinases [35, 36]. PMA stimulates PKC activity, while CI raises the intracellular level of Ca^2+^ [37, 38]. Many clinical studies have shown the upregulation of cytokines released (Th1-related cytokines) upon PMACI stimulation, namely, the IL-2, IL-6, IL-17, IFN-γ, and TNF-α. However, these studies did not affect the expression of IL-4 and IL-10 [39–41]. Another point to note in future research is that the expressions of IL-10, IL-17, IL-22, IFN-γ, and TNF-α are higher in IMDM than RPMI-1640 upon PMACI stimulation due to the concentration of Ca^2+^. According to Zimmermann and colleagues (2015), IMDM consists of 1.49 mM of Ca^2+^, while RPMI-1640 contains 0.42 mM of Ca^2+^. Therefore, by increasing the Ca^2+^ concentration in RPMI-1640 to 1.5 mM, the expression of cytokines is comparable to IMDM. Vice versa, the reduction of Ca^2+^ concentration in IMDM resulted in a lowered cytokine expression. Thus, 1.5 mM of Ca^2+^ is optimal for maximal ionomycin stimulation in cells [42].

When compared between PMACI, lectin phytohaemagglutinin (PHA), LPS, Con-A, and pokeweed mitogen, PMACI expresses the most potent cytokine production in a short period without significant damage to the cells [43]. On the other hand, C48/80 acts as a “selective” mast cell activator by stimulating the trimeric G-proteins and activating phospholipase C and D pathways [44]. C48/80 activation can bypass Ca^2+^ and PKC signal transduction, thus beneficial when PMACI cannot be used [35].

## LAD2 cell line

Before the discovery of LAD cells, HMC-1 cells were the only cell culture available to researchers that resembled human mast cells. However, HMC cells’ usefulness is limited by two deficiencies—they are growth factor independent, and they degranulate inconsistently to IgE-dependent signals, possibly due to the variable expression of the FcεRI α-subunit [45]. Laboratory of Allergic Diseases 2 (LAD2) human mast cells were first discovered through a routine study of cells from bone marrow aspirates of a mast cell sarcoma/leukemia patient [45]. During this routine study, researchers discovered cultures of mast cells with functional FcεRI and FcγRI receptors that continue to proliferate in a stem cell factor-containing serum-free media. These cells resembled CD34^+^-derived human mast cells and responded to human recombinant c-kit receptor ligand stem cell factor (rhSCF) while sharing similar characteristics with LAD1 cells. Morphologically, LAD2 cells stained with acid toluidine blue and tryptase are oval or round nucleated cells with metachromatically staining granules, and they measured between 8 to 15 µm diameter. Under the electron microscope, they appeared as cells with rough surfaces and cytoplasmic projections [45]. LAD cells highly resemble mast cells as they expressed surface FcεRI, cluster of differentiation (CD) 4, 9, 13, 22, 45, 64, 71, 103, 117, 132, C–C chemokine receptor type 5 (CCR5), and C-X-C chemokine receptor type 4 (CXCR4) and CD14, 31 and 32 on a lesser degree. They can release histamine and β-hexosaminidase upon FcεRI aggregation. To date, LAD2 cells have been used to study mast cell proliferation, receptor expression, mediator release/inhibition during mast cell degranulation, and cellular signaling [19, 46]. These cells are also commonly used in studies involving MRGPRX2. The MRGPRX2 is expressed by mast cells and degranulates upon binding by different ligands; it is also engaged in pseudo-allergic reactions, chronic spontaneous urticaria, atopic dermatitis, and allergic asthma [9].

LAD2 cells degranulate well when stimulated but lack the ability to generate cytokines, as experienced by Rådinger et al. (2010). Apart from that, Rådinger et al. (2010) also noted the relatively slow growth rate of LAD2 cells—a doubling rate of approximately 2 weeks. Although LAD2 cells require a longer time to proliferate compared to some tumorigenic cells, which take 3 to 5 days, Kirshenbaum et al. (2003) believed that this longer duration allowed the cells to exhibit a more mature phenotype. Another potential drawback of LAD2 cells is excessive clumping when the cells are grown for a prolonged duration. The slower growth may hamper the cells’ responsiveness to biotinylated IgE/streptavidin crosslinking, thus reducing activation and degranulation. However, this could be easily overcome by maintaining the cell concentrations between 0.25–0.5 × 10^6^ cells/mL to reduce cell clumping and performing hemidepletions every 3–4 days as suggested by Rådinger et al. (2010) and to freeze down cells frequently and then thaw and expand a new stock culture yearly [45].

### Cell source and culture conditions of LAD2 cell line

**Fig. 3:**
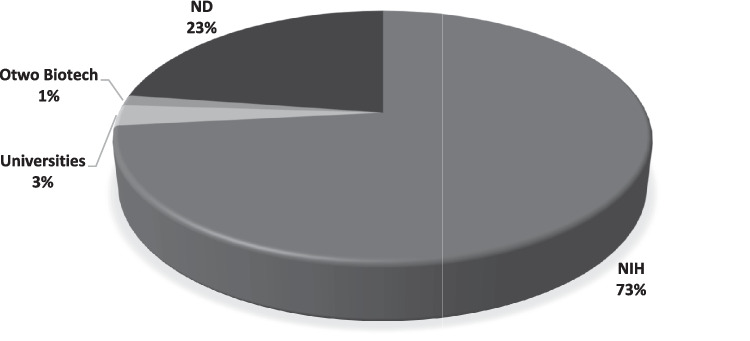
Sources of LAD2 cells. The proportion of articles using a particular source is indicated in the main text. LAD2 cells are commonly sourced from the National Institute of Health (NIH), USA, from the laboratory of Drs. Arnold Kirshenbaum and Dean Metcalfe. ND, no data

LAD2 cells are often sourced from the Laboratory of Allergic Diseases, National Institutes of Health (NIH) in Bethesda, United States of America (USA), where the cells were first discovered and successfully cultured. Of the 75 papers analyzed, 55 research groups obtained their LAD2 cells from NIH, specifically the laboratory of Drs Arnold Kirshenbaum and Dean Metcalfe [47]. Some were obtained from Otwo Biotech company (1 of 75), Dr. Yangyang Yu of Shenzhen University (1 of 75), Dr. Michael of Colombia University, and Professor Renshan Sun of Third Military Medical University (1 of 75). However, the authors did not report the source of their gifted LAD2 cell line. Finally, 17 research groups did not specify the origins of their LAD2 cells. Figure 3 summarizes the most common sources for obtaining LAD2 cells.

StemPro-34 is the medium used to culture LAD2 cells, as observed in most of the 75 papers analyzed (74 of 75). Only one publication reported the use of IMDM with supplementation of 10% FCS to culture the LAD2 cells. StemPro-34 is a specifically formulated serum-free medium used to support the growth of human hematopoietic cells. Supplements are often added in the StemPro-34 culture medium for LAD2 cells. The medium is usually supplemented with a StemPro-34 nutrient supplement, which includes pre-mixed penicillin/streptomycin, L-glutamine, and human stem cell factor. Apart from that, Zou et al. (2022) added 50 ng/mL of IL-6 into their StemPro-34 medium. Cytokines and growth factors are added to the medium to further support the progenitor cells’ growth. The use of StemPro-34 is in accordance with the medium used when LAD2 cells were first cultured in the laboratory of Drs. Kirshenbaum, Akin, and Metcalfe. Similarly, the medium was supplemented with 2 mM L-glutamine, 100 IU/mL penicillin, 50 µg/mL streptomycin, and 100 ng/mL rhSCF. Adding 100 ng/mL of rhIL-6 and 30 ng/mL of rhIL-3 for the first week was optional [45]. Kirshenbaum et al. (2003) also noted that even though LAD2 cells could survive in medium without SCF, the numbers doubled in approximately 3 weeks with 100 ng/mL SCF, and the addition of rhIL-3, rhIL-5, or rhIL-6 did not influence the cell numbers. SCF is needed to grow LAD2 cells as they do not possess the *c-kit* activating mutation at codon 816. LAD2 cells were grown in a humidified incubator at 37 °C with 5% CO_2_. Table 3 summarizes the culture medium used to grow LAD2 cells. As suggested by Rådinger et al. (2010), hemidepletions of the medium were performed by most researchers when growing the LAD2 cells at the frequency of once a week. The cell density was generally maintained between 1 × 10^5^ and 2 × 10^6^ cells/mL.

**Table 3:** Detailed information on the type of basal media, supplements used, and their respective number of articles for culturing LAD2 cells

Basal media		Supplements			
		Serum		Others	
Name	# articles	Name	# articles	Name	# articles
StemPro-34 IMDM	73 1	10% FCS	1	StemPro-34 nutrient	73

### Inducers and mediators release for LAD2 cells

As LAD2 cells express the high-affinity IgE receptor, they can be sensitized with antibodies to induce degranulation. Among the 75 shortlisted articles, only 24 reported that they sensitized LAD2 cells with the IgE antibody. Other studies directly activate the LAD2 cells to degranulate using their chosen inducer(s). Several inducers have been used in studies involving LAD2 cells. This includes the common C48/80 used by 36 of the studies. C48/80 is a known mast cell degranulator used in countless allergy-related studies [48]. Substance P is also commonly used as an inducer of LAD2 cells (14 of 75). Substance P is an undecapeptide found in the human body and secreted from specific sensory nerve terminals [49]. It is a known ligand of MRGPRX2 [50]. PAMP (9–20) is a significant product of the adrenomedullin precursor and was used by three research groups to induce LAD2 cells in this analysis [49]. Other inducers used to activate LAD2 cells include Tween 20, LL-37, (R)-ZINC-3573, CST-14, thapsigargin, and complement C3a, as shown in Table 4. Apart from these common inducers, several studies have analyzed the degranulation effects of antidepressants (clomipramine, paroxetine, and desipramine), contrast media (gadopentetate meglumine, iodinated contrast media, and iopamidol), p-Phenylenediamine (PPD), P17, morphine derivatives (thebaine and pethidine), fluoroquinolones, and many others. PPD is a component found in hair dyes implicated to induce immediate allergy, acute dermatitis, and contact dermatitis.[51]. On the other hand, P17 is a short host defence peptide from the ant *Tetramonium bicarinatum* venom [52]. LAD2 cells are commonly used to study MRGPRX2-related pseudo-allergy reactions, and thus, they were used to identify several novel mast cell compounds [53]. Besides that, the effects following exposure to influenza A virus were also studied using LAD2 cells [54]. As LAD2 cells are mast cells, some researchers pre-sensitize them before inducing them with the corresponding inducers. These studies used DNP-IgE as sensitizer (18 of 75) and then followed by either DNP-HSA, DNP-BSA, DNP-streptomycin, or streptavidin as inducers in their studies. Other combinations such as biotinylated IgE with streptavidin and human myeloma IgE with anti-IgE were also used by several researchers.

**Table 4:** Detailed information on the number of articles for the type of inducers, their induction time, and the type of mediators studied in LAD2 cell activation

Type inducers	# articles	Induction		Type of mediators	
Name		Time	# articles	Name	# articles
Substance P	14	< 1 h	60	β-Hexosaminidase	62
Tween 20	1	2**–**4 h	6	Histamine	36
PAMP (9–20)	3	5**–**8 h	30	5-HT	3
PAMP-12	1	13**–**24 h	13	Tryptase	6
Compound 48/80	36	2**–**4 days	3	CD63	3
LL-37	4	Unknown	5	TNF-α	28
(R)-ZINC-3573	3			IL-1α	1
CST-14	1			IL-1RA	1
Thapsigargin	1			IL-1β	2
Complement C3a	3			IL-2	1
Antidepressants	1			IL-4	3
Gadopentetate meglumine	1			IL-5	2
Iodinated contrast media	1			IL-6	5
p-Phenylenediamine	1			IL-8	27
P17	1			IL-10	2
Morphine derivatives	1			IL-13	5
IL-17A	1			IL-17	1
IL-33	3			IL-17A	2
FluA virus	1			IL-22	1
Novel mast cell compounds	1			IL-31	1
Mastoparan	1			IL-33	1
PMA and ionomycin	2			MCP-1	30
PMACI	1			CCL-1	1
Thimerosol	1			CCL-2	25
Iopamidol	1			CCL-3	2
Isosalvianolic acid C	1			CCL-4	3
Fluoroquinolones	1			CCL-5	3
Polymycin B and E	1			MIP-1α	2
Microvesicles from activated T cells	1			MIP-1β	3
SQ21 or SQ22	1			PGD2	6
Nanoparticle formulations	1			LTB4	1
Imidazolidinyl urea	1			GM-CSF	3
A23187	3			G-CSF	1
Biotin-conjugated IgE	1			Flt-3L	1
Water	1			CXCL-10	1
DNP-BSA	3			IFN-γ	2
DNP-HSA	4			FGF2	1
DNP-streptomycin	1			VEGF	1
Streptavidin	11			CysLTs	1
Human Astressin 2B	1				
WZ3146/PP2	1				
Anti-IgE	5				
NECA	1				
Milk particle	1				
IFN-β1	1				
Polypeptide	1				
Frozen and thawed 3 times	1				

*NECA*, 5′-N′(ethylcarboxamido)adenosine; *CysLTs*, cysteinyl leukotrienes

From the articles analyzed, histamine (36 of 75) and β-hexosaminidase (62 of 75) are the two common mediators that were often evaluated. Both mediators are proinflammatory mediators that play crucial roles as degranulation biomarkers [55]. Other mediators include cytokines and chemokines. Thirty out of 75 articles evaluated the levels of MCP-1, while 28 analyzed TNF-α. Several ILs were analyzed, including IL-8 (27 of 75) and IL-6 (5 of 75). Several other mediators evaluated include 5-hydroxytryptamine (5-HT), tryptase, MIP-1α, MIP2, prostaglandin 2 (PGD2), leukotriene B4 (LTB4), and granulocyte–macrophage colony-stimulating factor (GM-CSF). The induction time differs based on the type of mediators studied. From our analysis, generally, LAD2 cells were induced between 0 and 1 h when β-hexosaminidase and histamine were to be evaluated. The duration of 30 min (min) was the most common induction period, while some groups induced the cells for 40 min, with the longest being 2 h [56]. The shortest duration used to stimulate LAD2 cells to produce β-hexosaminidase was 15 min by Park et al. (2019) and histamine for 10 min by Sun et al. (2021) [57, 58]. For the evaluation of late mediators such as TNF-α, ILs, and MCP-1, longer induction time was needed, ranging from 6 to 8 h, with 6 h being the most used induction time. Two other induction times were noted which were 12 h and 24–48 h. In the influenza A virus study, the cytokines and chemokines were analyzed on days 1, 2, and 4 (maximum) post-infection [54] In another 12 h induction time study, the researchers evaluated the effects of substance P and PAMP (9–20) on LAD2 cells and analyzed the TNF-α, MCP-1, IL-8, and IL-31 levels [49]. On the 24–48 h induction time, the study studied the effects of P17 on LAD2 cells, analyzing MCP-1 and MIP-1α release [52]. Table 4 summarizes our analysis of the type of inducers, induction time, and type of mediators often used and studied by researchers using LAD2 cells.

## RBL-2H3 cell line

In mast cell research, the most common animal cell line used is the RBL-2H3 cell line [59]. The RBL cells were generated from rats injected with the chemical carcinogen β-chlorethylamine to induce basophilic leukemia [60]. The RBL cells were adapted to suspension cell culture (named RBL-1) and can specifically bind IgE to their surface membrane [61, 62]. Nevertheless, neither an IgE/antigen trigger nor chemical stimulation by a Ca^2+^ ionophore was able to cause RBL-1 to release histamine [63]. A responsive subline known as RBL-2H3 that degranulated in response to an IgE trigger was successfully isolated from subsequent cloning of RBL cells in 1981 [64]. These cells were a great model for understanding the FcεRI signaling cascades. They could be cultured in huge numbers to examine the characteristics and binding properties of IgE, leading to the signaling pathways involved in degranulation [65].

RBL-2H3 cells were widely employed as a mast cell model shortly after they were formed. Unquestionably, RBL-2H3 cells have the advantage of being a simple cell line to cultivate due to their short doubling time (18–24 h), enabling researchers to easily obtain a high number of homogenous cells. However, their suitability and credibility were eventually questioned [66, 67]. In the early years of establishing RBL cells, a variant with impaired cromoglycate binding had been identified in a population of RBL-2H3, suggesting that the cell line may not be entirely homogenous [68]. Although research reports frequently referred to RBL cells as mast cell lines, it was derived from basophils. Additionally, the cell line lacked consistency in the physiology of basophils or mast cells, and findings between other research groups utilizing the same cell line varied [66]. Mast cells and basophils are two different but functionally related cell types essential in type I hypersensitivity. Granulocyte basophils circulate, whereas mature mast cells are only found in tissues at the interfaces with the external environment, such as the lungs, skin, and mucosal surfaces [69]. Despite their similarities, it was suggested that the two cell types descended from distinct lineages [70, 71]. Numerous arguments even suggest they may share a common origin [72, 73].

Nevertheless, RBL-2H3 cells have been successfully used in investigations on IgE binding to FceRI receptors and subsequent downstream processes [66, 74]. The expression of rat mast cell protease II (RMCP-II) [75] and a similar expression of the c-kit receptor tyrosine kinase [76] in RBL-2H3 cells comparative to human HMC-1 and murine P-815 mast cells were one of the few aspects of mast cell physiology that supports the notion that RBL-2H3 cells can model mast cells. Moreover, numerous studies have found similarities between the exocytosis mechanisms of bone marrow mast cells and RBL cells involving SNARE proteins, designating RBL cells as a suitable model for research on MC exocytosis [59].

### Cell source and culture conditions of RBL-2H3 cell line

**Fig. 4:**
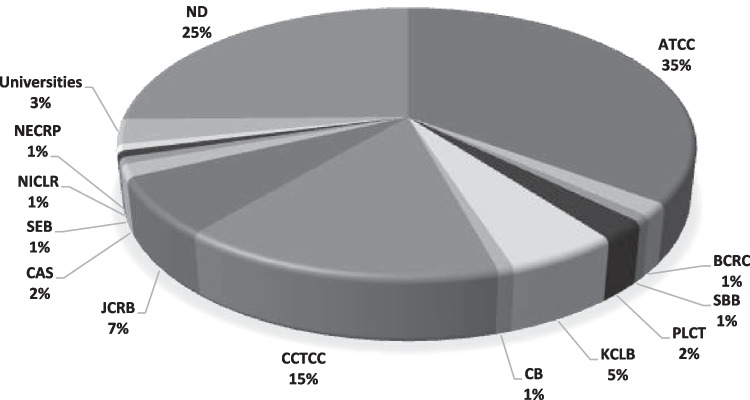
Sources of reported RBL-2H3 cell line. The proportion of articles using a particular source is indicated in the main text. ATCC, American Type Culture Collection; CCTCC, China Center for Type Culture Collection; JCRB, Japanese Collection of Research Bioresources Cell Bank; KCLB, Korean Cell Line Bank; BCRC, Bioresource Collection and Research Center; PLCT, Procell Life Science & Technology; NECRP, National Experimental Cell Resource Platform; SBB, Shanghai Binsui Biotechnology; CB, Cellcook Biotechnology; SEB, Shanghai EK-Bioscience Biotechnology; CAS, Chinese Academy of Sciences; NICLR, National Infrastructure of Cell Line Resource; ND, no data

Most of the RBL cell lines used in allergy research were the RBL-2H3 cell line (131 of 134), followed by humanized RBL cells (3 of 134). The American Type Culture Collection (ATCC) is the most reported source (46 of 131) for the RBL-2H3 cell line. Other cell bank sources of RBL-2H3 includes China Center for Type Culture Collection (CCTCC) (20 of 131), Japanese Collection of Research Bioresources Cell Bank (JCRB) (9 of 131), Korean Cell Line Bank (KCLB) (7 of 131), Procell Life Science & Technology (PLCT) (3 of 131), Bioresource Collection and Research Center (BCRC) (2 of 131), Chinese Academy of Sciences Shanghai (CAS) (2 of 131), Binsui Biotechnology (SBB) (1 of 131), Cellcook Biotechnology (CB) (1 of 131), Shanghai EK-Bioscience Biotechnology (SEB) (1 of 131), National Infrastructure of Cell Line Resource (NICLR) (1 of 131), and National Experimental Cell Resource Platform (NECRP) (1 of 131). Additionally, cells were received as gifts from scientists from other universities (4 of 131). However, the authors did not report the source of their gifted RBL cell line. Finally, 33 out of 131 publications did not specify the cell origin. Figure 4 summarizes the cell line sources of RBL-2H3.

ATCC and BCRC recommend RBL-2H3 to be cultured in MEM that contains Earle’s Balanced Salt Solution, non-essential amino acids, 2 mM L-glutamine, and 1 mM sodium pyruvate, made complete with the supplement of 15% FBS. CCTCC and JCRB recommend using the same MEM base media with a slight reduction of FBS to 10%. KCLB, however, recommends DMEM with 10% FBS. For institutions other than those mentioned above, there is no information regarding the recommended media used for the culture of RBL-2H3.

A review of the published methods revealed significant variation in the culture media and supplements used to cultivate RBL-2H3 cells (Table 5). However, 11 of 131 studies did not disclose the culture conditions used. DMEM was the most used (69 of 131), followed by MEM (44 of 131), MEM Eagle-alpha modification (2 of 131), RPMI-1640 (3 of 131), and EMEM (1 of 131). For serum, media were mainly supplemented with 10% FBS (91 of 131), followed by 15% FBS (26 of 131), 20% FBS (1 of 131), 17% FBS (1 of 131), and 5% FBS (1 of 131). Other supplements reported include penicillin/streptomycin (102 of 131), L-glutamine/GlutaMAX (16 of 131), sodium pyruvate (14 of 131), and non-essential amino acid (NEAA) (7 of 131). Two studies utilized distinctive medium formulation for the culture of RBL-2H3 cells: a concoction of 70% α-MEM, 20% RPMI 1640, and 10% FBS with 2 mM L-glutamine, as well as a mix of 41.5% MEM, 41.5% IMDM, and 17% FBS with 25 mM HEPES and 120 μg/mL gentamicin sulphate. There is yet to be any report on the influence of various media and supplements on RBL-2H3 cells’ physiology and function.

**Table 5:** Detailed information on the type of basal media, supplements used, and their respective number of articles for culturing RBL-2H3

Basal media		Supplements			
		Serum		Others	
Name	#articles	Name	#articles	Name	#articles
DMEM	69	10% FBS	91	Penicillin/streptomycin	102
MEM	44	15% FBS	26	L-glutamine/GlutaMAX	16
α-MEM	2	20% FBS	1	Sodium pyruvate	14
RPMI	3	5% FBS	1	NEAA	7
EMEM	1	17% FBS	1		
Mix α-MEM and RPMI	1				
Mix MEM and IMDM	1				

*DMEM*, Dulbecco’s Modified Eagle Medium; *MEM*, minimum essential media; *α-MEM*, Minimum Essential Media Eagle–alpha modification; *EMEM*, Eagle’s Minimal Essential Medium; *RPMI-1640*, Roswell Park Memorial Institute 1640; *IMDM*, Iscove’s Modified Dulbecco’s Medium; *FBS*, fetal bovine serum; *NEAA*, non-essential amino acids

### RBL-2H3 cell sensitization, induction, and degranulation

Following the crosslinking of their IgE-bound FceRI by multivalent allergens, RBL-2H3 cells, like mast cells and basophils, respond with degranulation, releasing a variety of mediators that trigger a potent immunological allergic response [77]. The cells demonstrated a bell-shaped dose–response to anti-DNP IgE, as observed in primary mast cells [78, 79]. A range of non-immunological triggers, such as the C48/80 and A23187, can also cause RBL-2H3 cells to degranulate [80]. Calcium ionophore A23187 can induce mast cell degranulation by increasing calcium entry into the cells through pore formation or as a transporting carrier [81]. RBL-2H3 cells behave similarly to basophils and mast cells when exposed to the A23187 [82, 83]. A23187 at a concentration of 5 µg/mL caused RBL-2H3 cells to degranulate about 50% of available histamine, which is 1.65 × more than that of IgE [67]. On the other hand, degranulation of MCs, especially connective tissue MCs, can be induced by polybasic compounds like C48/80. C48/80 can interact with G_0_ and G_i_ and their subfamilies of G-protein coupled receptors (GPCRs) to activate their downstream signaling of degranulation [84–86]. However, polybasic compounds were inactive on some MC subfamilies, including RBL cells, which may be due to their lack of G_i-3_ [87, 88]. Nonetheless, they can develop sensitivity to C48/80 depending on the culture conditions. Interestingly, quercetin can cause a rise in the expression of G_i-3_’s subunits, triggering RBL-2H3 response to C48/80 [88]. Another study also reported that RBL-2H3 cells change into a C48/80 active phenotype when co-cultured with 3T3 fibroblast [89]. Unfortunately, they were unable to ascertain the factors implicated in this phenomenon.

A review of the assays performed using RBL-2H3 cells (Table 6) demonstrated that most of the studies sensitize the cells with DNP-BSA and later induce the cells with DNP-HSA (91 of 131) and measured the degranulation of β-hexosaminidase (115 of 131) and histamine (61 of 131). The induction times were mainly reported to be within 1 h (91 of 131), with most studies reportedly inducing RBL cells for a full 1 h (29 of 131). One study used RBL-2H3 cells for cytotoxicity assay but did not investigate any mediators released nor used any inducers. β-Hexosaminidase is an exoglycosidase enzyme that is associated with degranulation, and it is released together with histamine [80]. Based on our review, β-hexosaminidase is the most common marker used to measure degranulation for RBL-2H3 cells. On the other hand, histamine has a significant role in allergy and inflammatory reactions as it mediates the interactions between inflammatory cells [90]. The pre-stored histamine within the granules of RBL-2H3 cells was inconclusive as a wide range of histamine content had been reported per 1 × 10^5^ cells: from 20–45 ng [91] to 100 ng [92] and up to 700 ng [67]. MCs have been known to produce proinflammatory cytokines like IL-4, IL-13, and TNF-α through the induction of TLR4 and TLR2 pathways [93]. RBL-2H3 has been observed to release up to 180 pg/mL of IL-13 and 60 ng/mL of TNF-α [94]. Our review revealed that 48 of 105 publications investigated and demonstrated cytokine production in RBL-2H3 cells. However, some studies reported conflicting findings that RBL-2H3 cells did not respond to lipopolysaccharide induction [66, 67]. Those studies also demonstrated that RBL-2H3 cells did not expressed CD14 and MyD88 that is implicated in TLR4 signaling pathway. They also reported that RBL-2H3 cells were also unresponsive to TLR2 ligands due to the lack of TLR2 receptors [66, 67].

**Table 6:** Detailed information on the number of articles for the type of inducers, their induction time, and the type of mediators studied in RBL-2H3 cell activation

Types of inducers		Induction		Type of mediators	
Name	# articles	Time	# articles	Name	# articles
DNP-HSA	91	< 1 h	91	β-Hexosaminidase	115
A23187	10	2–4 h	5	Histamine	61
C48/80	15	5–8 h	18	TNF-α	41
DNP-BSA	9	9–12 h	2	IL-1β	6
Substance P	3	13–24 h	19	IL-2	1
PMA	4	Unknown	10	IL-3	2
Patient Sera	2			IL-4	40
TNP-HSA	1			IL-5	4
TNP-BSA	1			IL-6	153
TNP-OVA	1			IL-8	6
Shrimp TM	1			IL-10	4
Oyster TM (Cra g 1)	1			IL-13	8
HDM	1			IL-18	2
Sodium sulfite	1			IFN-γ	5
PM2.5 suspension	1			Prostaglandin D2	4
Sodium metabisulfite	1			Prostaglandin E2	1
Unknown	1			MCP-1	2
				VEGF	1
				sICAM-1	1
				COX-2	1
				LTC4	4
				5-HT	1
				CCL-2	2
				CCL-17	1
				CCL-22	1
				CCL-25	1
				Tryptase	1

*DNP*, 2,4-dinitrophenyl; *BSA*, bovine serum albumin; *HSA*, human serum albumin; *PMA*, phorbol-12-myristate-13-acetate; *TNP*, trinitrophenyl; *TM*, tropomyosin; *HDM*, house dust mite; *PM*, particulate matter; *TNF*, tumor necrosis factor; *IL*, interleukin; *IFN*, interferon; *MCP*, monocyte chemoattractant protein; *VEGF*, vascular endothelial growth factor; *sICAM*, soluble intercellular adhesion molecule; *COX*, cyclooxygenase; *LTC*, leukotriene; *5-HT*, 5-hydroxytryptamine; *CCL*, CC motif chemokine ligand

### Humanized RBL cells

As discussed above, RBL cells originated from rats. It was established that rat IgE can bind to human IgE receptors [95]. However, rat IgE receptors did not recognize human IgE [96]. RBL cells expressing the high-affinity IgE receptor FcεRI implied that the cell line may be suitable for detecting allergen-specific IgE and allergens [59]. Stable transfection of the human FcεRI α chain in RBL cells was achieved in the 90 s with the receptor-expressed mediated antigen-induced signaling and mediator release [97–100]. These humanized RBL cells were then utilized to investigate the ability of pure peanut allergens and other food allergens to induce degranulation [101–104]. RBL-SX38 is one of the commonly used humanized RBL cell lines that express α, β, and γ chains of human FcεRI [105].

Adding a reporter gene (firefly luciferase) to humanized RBL cells paved the way for straightforward and susceptible detection of cellular activity via the IgE receptor [59]. The first and most common system was a nuclear factor of activated T-cells (NFAT)-firefly luciferase reporter that is linked to IgE-dependent signal transduction and is named RS-ATL8 cells [106]. The calcium influx from stimulation of the cells activates calcineurin, a phosphatase that dephosphorylates NFAT. This unveiled the nuclear localization signal in the N-terminal and led to nuclear translocation. NFAT then attaches to specific promoter regions and initiates gene transcription of the luciferase reporter gene [107]. Activation of these cells may be detected by detecting luciferase activity with suitable substrates and a luminescence detector [106]. RS-ATL8 assay is very sensitive and well suited for high throughput format. The assay has since been employed to identify food or other allergens and determine the allergenicity of vaccines [108–110].

Our literature retrieval identified two articles using the humanized RBL cells, RBL-SX38 [111, 112], and one article using the reporter humanized cells, RS-ALT8 [113], to investigate the release of β-hexosaminidase. RBL-SX38 was obtained from a research group at Harvard Medical School, USA, while the study using RS-ALT8 did not specify the cells’ origin. RBL-SX38 and RS-ALT8 cells were grown in MEM supplemented with 10% FBS, L-glutamine, and antibiotics. One study using RBL-SX38 did not specify the culture condition. In all three studies, cells were sensitized with allergen-specific human IgE and induced by their respective allergens.

## Alternative mast cell lines for in vitro studies

Apart from the mentioned cell lines, there are other alternative options for immortalized in vitro mast cell lines in allergy studies, albeit not as commonly reported. One of them is the Immortalized Human Mast Cell Line (LUVA). Laidlaw and her colleagues first identified and characterized this cell line in 2011 [114]. The cells were grown from CD34 + -enriched mononuclear cells derived from the peripheral blood of a donor with aspirin-exacerbated respiratory disease [114]. The authors mentioned that this cell line can be maintained without stem cell factors to survive and proliferate without further addition of any growth factors for approximately 2 years. In addition, LUVA cells also display high levels of c-kit, metachromatic cytoplasmic granules, and FcεRI [114]. These cells will prove valuable for functional human mast cell studies as they can be induced to degranulate and release various allergic mediators such as β-hexosaminidase, prostaglandin D2, thromboxane A2, and MIP-1β.

Another human mast cell line suitable for allergy studies is the ROSA^KIT^ mast cells. This cell line was first reported in 2014 [115]. Unlike LUVA cells, these cells require stem cell factor to survive and proliferate with a doubling time of 24 h [115]. ROSA^KIT^ cell also has functional IgE receptors, allowing it to be easily activated to release mediators whenever FcεRI crosslinking happens [115]. Apart from activation through IgE receptors, this human mast cell line can also be induced using calcium influx. A reported study showed that the ROSA^KIT^ cell induction using calcium ionophore A23187 was able to release 80% of β-hexosaminidase as compared to using IgE-FcεRI, which only releases approximately 38% of the enzyme after 1 h induction [115].

Additionally, immortalized mast cell lines from mouse origin, such as MC/9, P815, and NCL-2, are also available. The MC/9 is a cloned mast cell line derived from the fetal liver of a (B6 × A/J)F1 mouse [116]. These cells can be sensitized to specific antigens by incubating them with IgE having the desired antigenic specificity, resulting in the release of soluble mediators such as histamine and leukotrienes when induced [117]. A reported study by Jin et al. (2019) has also induced these cells using PMA (50 nM) and A23187 (1 μM) for 24 h to measure the release of histamine and LTC4 levels [118]. P815 cells, on the other hand, were isolated from a mouse with mastocytoma in 1957 [118, 119]. These cells do not express the IgE high-affinity receptor and, therefore, can only be activated independent of the IgE pathway. However, they express fragment crystallizable gamma receptor II (FcγRII). They could bind the Fc portion of mouse IgG antibodies through their fragment antigen-binding (Fab), which may recognize NK cells activating receptors and lead to target cell lysis [120]. In addition, one study reported using C48/80 and CI to induce degranulation in P815 cells to release allergic mediators such as IL-4 and histamine [121]. The NCL-2 cell line was an immortalized mast cell derived from the bone marrow of a male NC/Nga mouse. This cell line does not carry a Kit mutation and could be maintained without additional growth factors except for FCS [122]. Hence, it could respond well to exogenous growth signals such as SCF and IL-3 and release various chemical mediators against several stimuli, including crosslinking of FcεRI [122]. Using this cell line will lead to an easier analysis of normal mast cells’ proliferation, differentiation, and function.

Finally, there are also other in vitro mast cell lines that are commonly used as an allergy model for studies of the role of mast cells in health and diseases, such as the bone marrow-derived mast cells (BMMCs), peritoneal mast cells (PMCs), and CD34( +) pluripotent hematopoietic derived human mast cells (HuMCs). However, these cell lines are primary cell lines isolated from the tissue and not modified. Hence, they have a definite life span. Due to this reason, these cell lines are not discussed extensively in this systematic review.

## Conclusion

Using secondary mast cell lines to conduct allergy research has come a long way since the discovery of mast cells in 1863 [123]. Today, in modern research, the scientific community can utilize different mast cell lines to study their role as a critical contributor in developing and propagating hypersensitivity reactions, as well as innate and adaptive immune responses [124]. This systematic review illustrated the popular immortalized mast cell lines used to study the molecular and cellular mechanisms involved in functional studies in allergy diseases and to determine potential protective compounds for allergic treatments. Therefore, some of these well-known cell lines, such as RBL-2H3, HMC-1, and LAD2 cells, have been proven to be an asset in unravelling the molecular complexity of allergy. However, every cell line will have its drawbacks, such as the absence of high-affinity IgE receptors and long doubling time (Table 7). In addition, the differences in cell source and culture maintenance may influence these cells’ epigenetic character, potentially leading to variations in results and inconsistency across laboratories. Thus, researchers must consider these factors before selecting the appropriate mast cell line for their studies.

**Table 7:** Summary of characteristics between RBL-2H3, LAD2, and HMC-1 cell lines

	Cell line		
	RBL-2H3	LAD2	HMC-1
Cell line origin	Wistar rat basophil cell	Bone marrow of an MCL patient	Peripheral blood of an MCL patient
Year of its first description	1978	2003	1988
Cell morphology	Fibroblast shaped cells	Round or irregularly shaped cells	Oval or irregularly shaped cells
Adherent/suspension cell line	Adherent	Suspension	Suspension
Doubling times	18–24 h	2 weeks	24–72 h
Presence of FcεRI receptor	Yes	Yes	No
SCF dependency	No	Yes	No
c-KIT mutation	WT KIT	WT KIT	V560G KIT D816V KIT
Reported cell surface markers	CD14, CD63, CD117, CD284	CD4, CD9, CD13, CD14, CD22, CD31, CD32, CD33, CD34, CD45, CD63, CD64, CD71, CD103, CD117, CD132, CD133, CD184, CD193, CD195	CD2, CD9, CD13, CD24, CD31, CD32, CD33, CD44, CD45, CD48, CD50, CD52, CD54, CD58, CD59, CD63, CD66, CD69, CD71, CD87, CD95, CD117, CD164, CD166, CD184
Other known characteristics	lack consistency in the physiology of mast cells	lack the ability to generate cytokines when stimulated	lack of mature HuMCs markers (i.e. tryptase and chymase)

*MCL*, mast cell leukemia; *WT*, wild type; *HuMCs*, human mast cells

## Data Availability

Data available on request from the authors.
